# The multifaceted regulatory roles of Nudix hydrolases in cancer and their therapeutic potential

**DOI:** 10.3389/fonc.2025.1743098

**Published:** 2026-01-14

**Authors:** Jianguo Pan, Jiaxi Yang, Kewei Wang

**Affiliations:** Department of Gastrointestinal Surgery, the First Affiliated Hospital of China Medical University, Shenyang, Liaoning, China

**Keywords:** cancer, NUDT, oncogene, prognosis, proliferation

## Abstract

The NUDT family comprises evolutionarily conserved enzymes that hydrolyze diverse substrates, including nucleoside phosphates, inositol polyphosphates, and RNA caps. Contrary to earlier perspectives focusing primarily on genome protection, compelling evidence now indicates that the majority of NUDT function as pro-tumorigenic factors. Cancer’s complex landscape, characterized by uncontrolled proliferation, evasion of apoptosis, metabolic reprogramming (like the Warburg effect), and genomic instability, creates an environment where NUDT exert significant influence. Key NUDT members, such as MTH1 (NUDT1), NUDT5, NUDT15, and NUDT22, are frequently overexpressed in cancers and actively promote tumor survival and progression. They achieve this not only by “sanitizing” the nucleotide pool to maintain genomic stability in cancer cells (hydrolyzing damaged nucleotides/caps), but also by dysregulating critical signaling pathways. The relationship between NUDT and cancer is multifaceted, involving intricate roles in nucleotide metabolism, redox homeostasis, and DNA repair. This functional diversity underscores their potential as therapeutic targets. Pharmacological inhibition of specific NUDT, particularly MTH1 and NUDT5, is an active area of research. Such inhibition aims to exploit cancer cell vulnerabilities by increasing the accumulation of damaged nucleotides and enhancing susceptibility to DNA-damaging agents (e.g., chemotherapy, radiotherapy) or PARP inhibitors, offering promising avenues for novel combination therapies. This review comprehensively overviews the mechanisms, diverse functions, and pathophysiological roles of NUDT in cancer biology, critically evaluating their therapeutic potential and the challenges in targeting them.

## 1 Introduction

**Figure 1:**
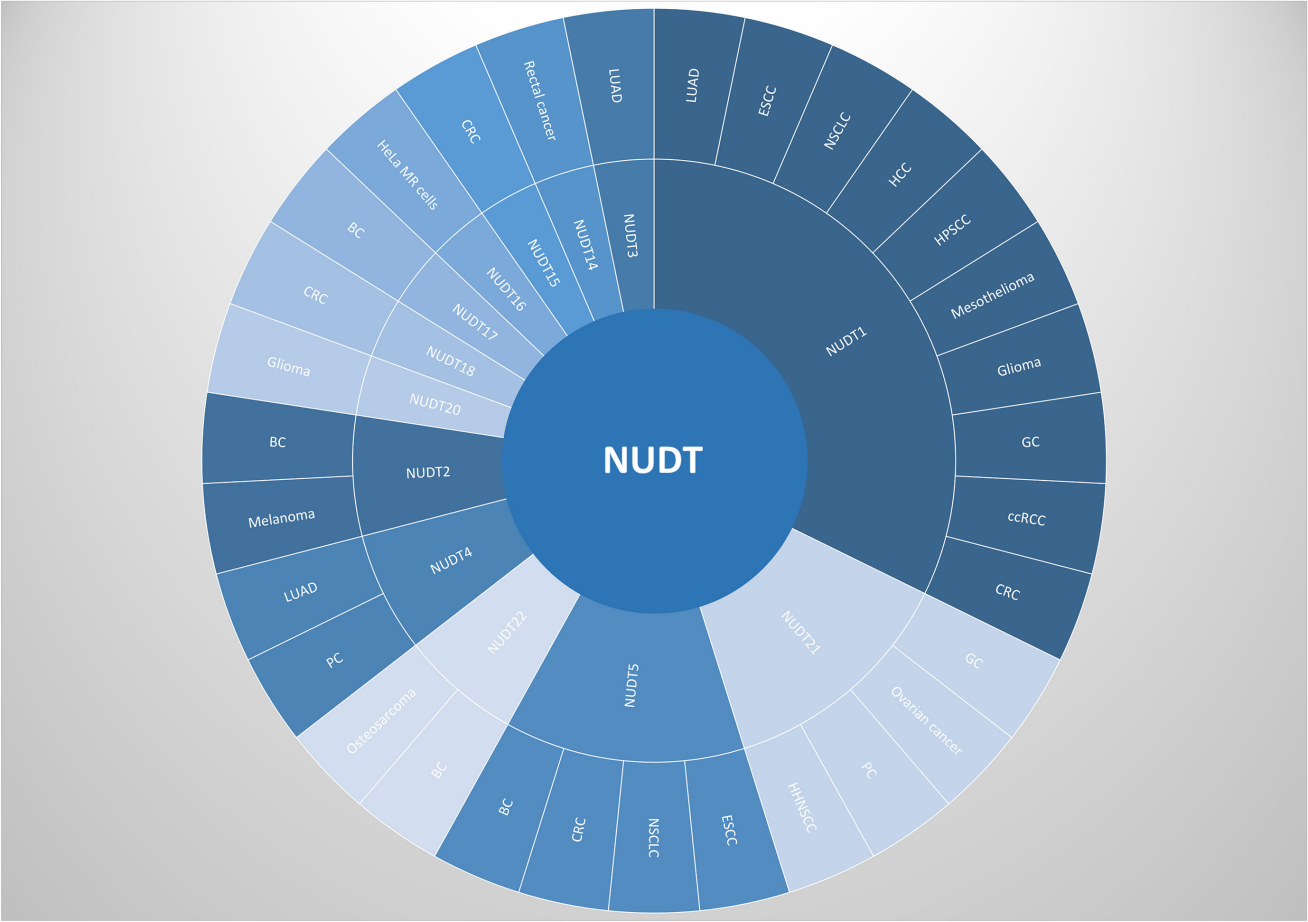
NUDTs currently known to play an important role in the occurrence and development of cancers. All these NUDTs in this figure play as oncogenes. CRC, colorectal cancer; ccRCC, clear cell renal cell carcinoma; GC, gastric cancer; HPSCC, hypopharyngeal squamous cell carcinoma; HCC, hepatocellular carcinoma; NSCLC, non-small-cell lung cancer; ESCC, esophageal squamous carcinoma; BC, breast cancer; PC, pancreatic cancer; HNSCC, head and neck squamous cell carcinoma;LUAD, lung adenocarcinoma.

**Figure 2:**
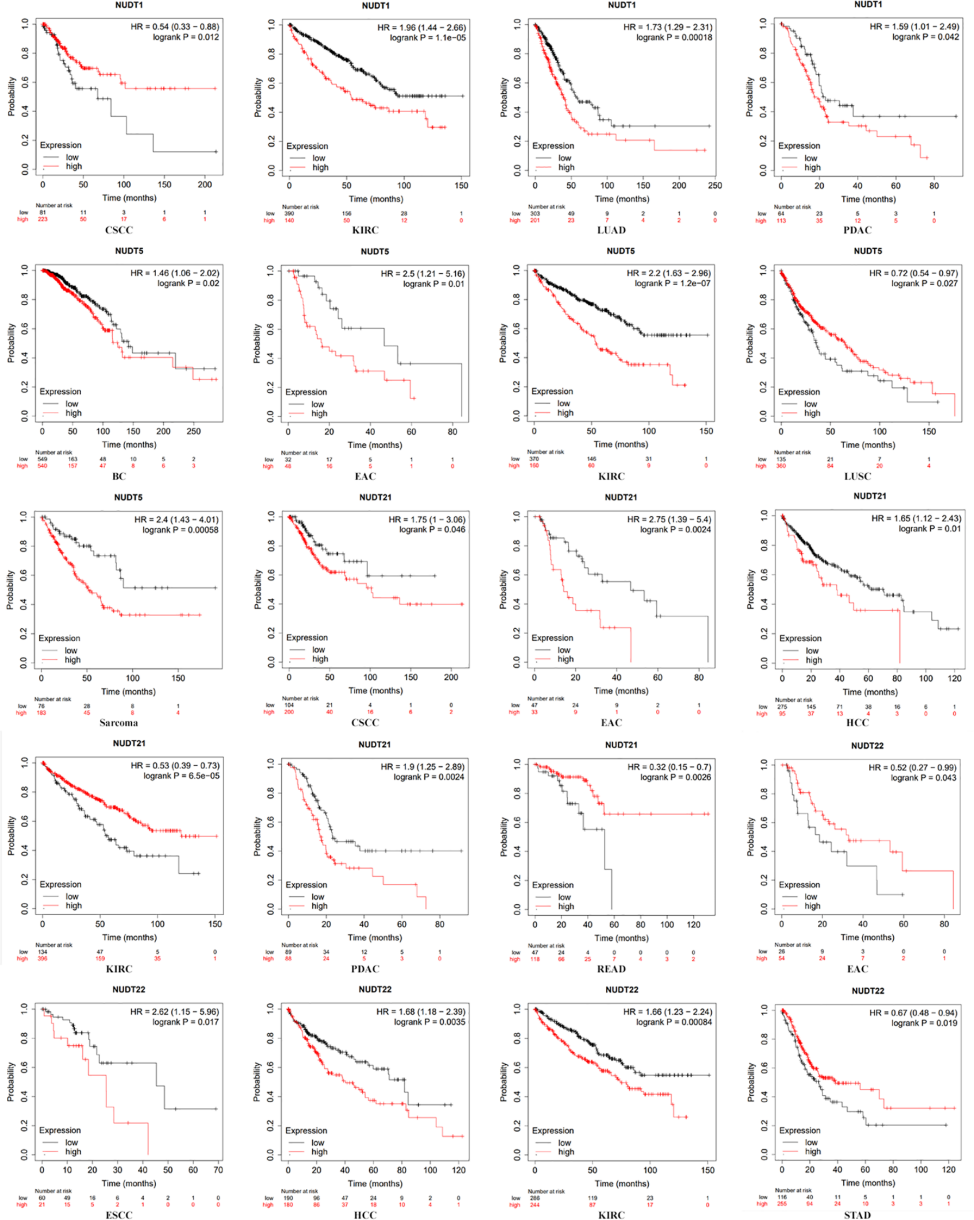
Correlation between the expression levels of NUDT and patient survival. Expression data were analyzed using KM plotter (http://kmplot.com/). Patients were split by median expression. HRs (hazardous ratios), 95% CIs (confidence intervals), and log-rank P-values are indicated. The relationship between the expression levels of NUDT1, 5, 21, 22 and prognosis of patients with BC (breast cancer)、CSCC (cervical squamous cell carcinoma)、EAC (esophageal adenocarcinoma)、ESCC (esophageal squamous cell carcinoma)、KIRC (kidney renal clear cell carcinoma)、HCC (hepatocellular carcinoma)、LUAD (lung adenocarcinoma)、LUSC (lung squamous cell carcinoma)、PDAC (pancreatic ductal adenocarcinoma)、READ (rectum adenocarcinoma)、STAD (stomach adenocarcinoma).

Nucleoside diphosphate-linked moiety X (Nudix) hydrolases (NUDT), a conserved enzyme family with pyrophosphorylase activity, regulate cellular metabolism and mRNA stability. The NUDT family critically impacts cancer biology through dual mechanisms. NUDT1 prevents DNA mutagenesis by hydrolyzing oxidized dNTPs, while NUDT2/3/12/15/17/19 regulate mRNA stability via decapping activity. Most members are upregulated in tumors (**Figure 1**), whereas tumor-suppressive isoforms show downregulation, reflecting their functional duality in oncogenesis. Dysregulated NUDT expression correlates with tumor progression, with select members (NUDT1/5/15) serving as chemotherapy targets or prognostic markers (**Figure 2**). This review synthesizes decade-long advances in NUDT family functions, emphasizing cancer mechanisms and clinical relevance of well-characterized members (**Table 1**).

**Table 1:** Potential functions of NUDTs in cancers.

Name	Alternative name	Related cancers	Upregulation	Downregulation	Prognostic predictors	Type	Functions	References
NUDT1	MTH1	CRC	✓		✓	*In vitro*	Promote cell proliferation, enhance activities of 8-oxo-dGTPase in CRC	(4, 5)
		ccRCC	✓		✓	*In vivo* and vitro	Promote the progress of ccRCC, reduce the sensitivity of ccRCC to sunitinib	(6, 7)
		GC	✓		✓	*In vitro*	Promote cell proliferation, invasion, migration and survival	(8, 9)
		Glioma	✓		✓	*In vivo* and vitro	Promote cell proliferation, colony formation, invasion, migration and tumor growth	(10, 11)
		Mesothelioma	✓		✓	*In vivo* and vitro	Promote cell proliferation and mesothelioma progression, therapeutic target	(12, 13)
		OSCC	✓		✓	*In vitro*	Determine the histopathological grades	(14)
		HPSCC	✓		✓	*In vivo* and vitro	Promote cell proliferation	(15)
		HCC	✓		✓	*In vitro*	Promote cell proliferation, invasion and migration	(16)
		NSCLC	✓		✓	*In vitro*	Promote cell proliferation, invasion, migration, inhibit apoptosis	(17, 18)
		LUAD	✓		✓	*In vitro*	Sustains oxidative DNA damage defense across resistance states.	(19)
		ESCC	✓		✓	*In vitro*	Promote cell proliferation, invasion, migration and EMT	(20)
NUDT2 NUDT3	Ap4A hydrolase 1	BC	✓		✓	*In vitro*	Promote cell proliferation	(28, 29)
		TNBC	✓			*In vitro*	Promote cell proliferation, invasion, migration	(30)
		Melanoma	✓		✓	*In vivo* and vitro	Promote anchorage-independent growth of cells, colony formation, migration, EMT and tumor growth	(32)
		LUAD	✓			*In vitro*	Promote lung adenocarcinoma progression	(87)
NUDT4	DIPP2	ccRCC		✓	✓	In silico	Influence the sensitivity of ccRCC cells to selumetinib	(48)
		LUAD	✓		✓	*In vitro* and silico	Promote cell proliferation	(88)
		PC	✓			*In vivo* and vitro	Exert as an oncogene, involved in the regulation of apoptosis	(89)
NUDT5	hYSAH1, YSA1H	BC	✓		✓	*In vivo* and vitro	Promote cell proliferation, invasion, migration, tumor growth and metastasis	(35–42)
		CRC	✓		✓	*In vitro*	Promote cell proliferation	(5)
		NSCLC	✓		✓	*In vitro*	Promote cell proliferation, invasion, migration, tumor growth and metastasis, inhibit apoptosis	(18)
		ESCC	✓		✓	*In vitro*	Promote cell proliferation, invasion, migration and EMT	(20)
		ccRCC			✓	In silico	Influence overall survival	(43)
NUDT7		CRC		✓		*In vivo* and vitro	Inhibit cell proliferation and tumorigenesis	(92)
NUDT10 NUDT13	DIPP3a, hDIPP3alpha	GC		✓	✓	*In vitro*	As a clinical diagnostic index	(46)
		Prostate cancer			✓	In silico	Reduce recurrence-free survival	(49)
		CRC		✓		*In vivo* and vitro	Inhibit initiation and proliferation.	(90)
NUDT14	UGPP	Rectal cancer	✓			In silico	Be related to tumorigenesis	(95)
NUDT15	MTH2	Leukemia				*In vitro* and silico	Determine the dose and effectiveness of thiopurine	(51, 52, 55, 62)
		CRC	✓		✓	*In vitro*	Promote cell proliferation	(5)
NUDT16		T-ALL		✓		*In vitro*	Delay the development of disease	(60)
		HeLa MR cells				*In vitro*	Promote cell proliferation	(57)
NUDT17		ccRCC			✓	In silico	Influence overall survival	(43)
		BC			✓	In silico	Promote growth and metastasis	(93)
NUDT18	MTH3	CRC	✓		✓	*In vitro*	Promote cell proliferation	(5)
		EC	✓		✓	*In vitro* and silico	Inhibit cancer progression.	(91)
NUDT20	DCP2	Glioma	✓		✓	*In vitro*	Promote cell proliferation, invasion, and migration, inhibit apoptosis	(63)
		NSCLC				*In vivo* and vitro	Inhibit the carcinogenic factors WFDC21P and miR-4293	(64)
		SCLC		✓		*In vivo* and vitro	Reduce chemotherapy resistance	(65)
NUDT21	CFIm25	KIRC		✓	✓	*In vivo* and vitro	Inhibit cell proliferation and tumor formation	(75)
		CC		✓	✓	*In vivo* and vitro	Inhibit cell proliferation, migration, invasion, tumorigenesis and lung metastasis	(76)
		Bladder Cancer		✓	✓	*In vivo* and vitro	Inhibit cell proliferation, migration, invasion	(76)
		HCC		✓	✓	*In vivo* and vitro	Inhibit cell proliferation, migration	(78–80)
		BC		✓	✓	*In vitro*	Inhibit cell proliferation, invasion, migration and EMT	(81)
		SCLC		✓	✓	*In vivo* and vitro	Promote apoptosis, inhibit cell proliferation	(82)
		HNSCC	✓		✓	*In vitro* and silico	Promote cell proliferation, inhibit apoptosis, immunoregulatory functions and immune checkpoint effects	(70)
		PC	✓		✓	*In vivo* and vitro	Promote cell proliferation, colony formation, migration	(67–69)
		GC	✓		✓	*In vivo* and vitro	Promote cell proliferation, colony formation, invasion, migration	(71)
		Ovarian cancer	✓			*In vitro*	Promote cancer malignant progression	(72)
		Prostate cancer	✓			*In vivo* and vitro	Drive enzalutamide resistance	(74)
NUDT22		Osteosarcoma, BC	✓			*In vivo* and vitro	Promote cancer growth	(86)

CRC, colorectal cancer; ccRCC, clear cell renal cell carcinoma; GC, gastric cancer; OSCC, oral squamous cell carcinoma; HPSCC, hypopharyngeal squamous cell carcinoma; HCC, hepatocellular carcinoma; NSCLC, non-small-cell lung cancer; ESCC, esophageal squamous carcinoma; BC, breast cancer; TNBC, human triple negative breast cancer;CML, chronic myelogenous leukemia; LUAD, lung adenocarcinoma; PC, pancreatic cancer; EC, endometrial cancer; T-ALL, T-cell acute lymphoblastic leukemia; SCLC, small cell lung cancer; KIRC, kidney renal clear cell carcinoma; CC, cervical cancer; HNSCC, head and neck squamous cell carcinoma; B-ALL, B-cell acute lymphoblastic leukemia; EMT, epithelial-mesenchymal transition.

## 2 Roles of NUDT family members in cancer

### 2.1 NUDT1

NUDT1 (also known as MTH1), a key hydrolase, promotes oncogenesis by hydrolysing 8-oxo-dGTP, thereby preventing the incorporation of mutagenic 8-oxoG into DNA (1). Its enzymatic activity correlates with mRNA levels across malignancies (2). Furthermore, in MYC-driven cancers, the oncoprotein creates a therapeutic vulnerability by establishing a dependency on NUDT1, which sanitizes the nucleotide pool via Polo like kinase 1 (PLK1) signaling, a liability exploited by LC-1-40, a targeted degrader of NUDT1 (3). In colorectal cancer (CRC), elevated NUDT1 levels are associated with reduced recurrence-free survival (4, 5). Mechanistically, hypoxia-inducible factor 2alpha (HIF2α)-driven NUDT1 overexpression in clear cell renal cell carcinoma (ccRCC) mitigates oxidative stress, promoting tumor progression and sunitinib resistance. NUDT1 knockdown suppresses ccRCC growth *in vivo* (6). Clinically, NUDT1 expression correlates with advanced ccRCC stages and may predict immune checkpoint inhibitor responsiveness (7). Gastric cancer (GC) models reveal NUDT1’s oncogenic role through enhanced proliferation/migration, associating with deeper invasion and poor survival (8). In GC, the deubiquitinase USP9X stabilizes NUDT1 protein levels, directly promoting cancer cell proliferation, survival, and invasion (9).

NUDT1 demonstrates potent oncogenic activity across multiple malignancies. In gliomas, its overexpression at mRNA and protein levels drives tumorigenesis by enhancing proliferation, migration, and invasion (10), while silencing suppresses xenograft growth and malignant phenotypes (11). Mesothelioma exhibits elevated NUDT1 expression correlating with poor survival, where its inhibition reduces tumor viability and synergizes with anti-PD-L1 (programmed death-ligand 1) therapy (12, 13). NUDT1 serves as an independent prognostic biomarker in oral squamous cell carcinoma (OSCC), with expression levels linked to histopathological grade and survival outcomes (14). Hypopharyngeal squamous cell carcinoma (HPSCC) shows coordinated NUDT1/Fusobacterium nucleatum (Fn) interactions that amplify proliferation and autophagy. Genetic knockdown reverses these effects and attenuates *in vivo* tumor progression (15).

NUDT1 overexpression correlates with aggressive clinicopathological features in hepatocellular carcinoma (HCC), including grade, stage, size, differentiation, vascular invasion, overall survival (OS), and disease-free survival (DFS). Its inhibition suppresses colony formation, migration, and invasiveness, underscoring its prognostic and therapeutic relevance (16). Additionally, elevated NUDT1 expression in non-small-cell lung cancer (NSCLC) is associated with tumor metastasis and prognosis. The knockdown of NUDT1 significantly inhibits NSCLC cell proliferation, migration, and invasion, and reduces xenograft tumor growth and metastasis (17, 18). In lung adenocarcinoma (LUAD), oncogenic KRAS signaling upregulates NUDT1, and crucially, its essential 8-oxodGTPase activity is maintained via compensatory AKT signaling upon KRAS addiction loss, establishing it as a persistent therapeutic vulnerability (19). Similarly, high levels of NUDT1 expression in esophageal squamous carcinoma (ESCC) are typically associated with poor prognosis. Depletion of NUDT1 not only suppresses ESCC cell proliferation and delays the G1 phase of the cell cycle, but also inactivates the microtubule-associated protein kinase/methyl_ethyl_ketone/extracellular regulated kinase (MAPK/MEK/ERK) pathway, resulting in decreased migration and invasion of ESCC cells (20).

The role of NUDT1 in cancer treatment is becoming increasingly evident. TH588, a NUDT1 inhibitor, has been the subject of ongoing research since its discovery in 2014. As a direct active-site inhibitor, TH588 binds the NUDT1 nucleotide pocket, forming key hydrogen bonds with Asn33, Asp119, and Asp120. This binding occludes the catalytic site and inhibits the function of the essential catalytic residue Glu56, thereby blocking dNTP sanitation and promoting the incorporation of oxidized nucleotides into DNA (1). Concurrently, it acts as a microtubule-modulating agent, destabilizing spindle dynamics and activating the USP28–p53 mitotic surveillance pathway independently of NUDT1 inhibition (21). This dual mechanism underpins its efficacy in preclinical models, including reduced survival of colon and neuroendocrine cancer cells and sensitization to chemotherapy and radiotherapy (22, 23). Building upon this mechanistic insight, the clinical derivative of the mitotic MTH1 inhibitor, Karonudib (TH1579), which preserves the dual activity profile, has commenced clinical trials for hematological malignancies (24). Crizotinib, another small-molecule inhibitor of NUDT1, disrupts nucleotide pool homeostasis by inhibiting NUDT1, resulting in DNA damage in cancer cells and the suppression of tumor growth (25, 26).

Beyond promoting core oncogenic processes, NUDT1 critically contributes to therapeutic resistance and modulates the tumor microenvironment. It confers resistance to targeted therapies such as sunitinib in ccRCC by mitigating treatment-induced oxidative stress (6). Moreover, NUDT1 engages in dynamic crosstalk with components of the microenvironment, notably exemplified by its functional synergy with the tumor-associated bacterium Fn in HPSCC to enhance cancer cell proliferation and autophagy (15).

In summary, NUDT1 is a multifunctional oncoprotein that promotes tumor growth, therapeutic resistance and interactions between the tumor microenvironment and microbes. Disrupting nucleotide homeostasis with inhibitors such as TH588 and crizotinib triggers DNA damage and makes cancers more susceptible to chemotherapy and radiotherapy. In a clinical context, NUDT1 serves as an independent prognostic biomarker in CRC and ccRCC, correlating with aggressive progression. Due to its dual roles in cell proliferation and treatment resistance, NUDT1 is a pivotal combinatorial target for epithelial and glial malignancies, enabling strategies to overcome therapeutic barriers and suppress tumor formation.

### 2.2 NUDT2

NUDT2 is a hydrolase related to viral RNA degradation (27). While initially identified as an estrogen-suppressive gene, NUDT2 paradoxically enhances breast cancer (BC) proliferation via human epidermal growth factor receptor 2 (HER2) pathway, with elevated levels in invasive ductal carcinoma correlating with poor prognosis (28). NUDT2 functions as a novel positive regulator of the oncogenic mammalian target of rapamycin complex 1 (mTORC1) pathway. It facilitates the lysosomal translocation and activation of mTORC1 by binding to and regulating Rag GTPase. This NUDT2-mTORC1 signaling axis is critical for cancer cell proliferation. Consequently, NUDT2 silencing disrupts BC cell cycle progression (G0/G1 arrest) by downregulating cyclin D1 and retinoblastoma phosphorylation (29). Critically, NUDT2 is overexpressed in human triple negative breast cancer (TNBC) and drives its tumorigenicity, as its knockdown suppresses proliferation, cell cycle progression, migration, and invasion (30). Furthermore, NUDT2 promotes tumor growth, invasion, and metastasis in melanoma and chronic myelogenous leukemia (CML). Targeted inhibition suppresses migration, induces apoptosis, and reverses immunosuppression in these malignancies (31, 32). Collectively, these findings underscore the potential of NUDT2 as a chemotherapeutic target for human melanoma and CML. These findings position NUDT2 as a context-dependent therapeutic target, warranting further exploration of its tissue-specific regulatory networks and clinical potential in solid tumors and hematologic cancers.

### 2.3 NUDT5

The canonical role of NUDT5 is as an ADP-ribose hydrolase that supports DNA repair by modulating local nucleotide pools at damage sites. For cancer cells experiencing replication stress, this function becomes a critical dependency, positioning NUDT5 as a therapeutic target whose inhibition could disrupt repair and synergize with genotoxic therapies (33). NUDT5 drives BC progression via multifaceted oncogenic mechanisms. In hormone receptor-positive BC, it sustains tumor growth, maintains stemness, and regulates progesterone-dependent transcriptional programs, with TH5427 inhibiting proliferation by disrupting these pathways (34, 35). Beyond regulating hormone signaling, NUDT5 functions as a master upstream regulator that orchestrates the expression of multiple key oncogenic driver genes, including USP22, RAB35B, FOCAD, and PTGES. This regulatory capacity underpins critical cancer phenotypes such as cell adhesion, cancer stem cell maintenance, and epithelial-mesenchymal transition (EMT), thereby promoting metastatic potential and tumor aggressiveness. Notably, NUDT5 inhibition disrupts these pathways and prevents oncosphere formation in 3D culture models (36). Virtual screening identified NUDT5-targeting ligands for BC therapy (37), with TNBC studies showing NUDT5 upregulation protects against oxidative DNA damage, while its inhibition via TH5427 reduces DNA replication and tumor growth both *in vitro* and *in vivo* (38). Clinically, elevated NUDT5 expression correlates with poor prognosis in estrogen receptor-positive (ER+) BC, driven by protein kinase B (AKT)/cyclin D pathway activation, positioning it as a prognostic biomarker (39, 40). Therapeutic targeting of NUDT5 is advancing through structure-based drug discovery. Virtual screening identified iron (III) metal complexes (compounds 2 and 6) and coumarin derivatives as potent inhibitors, with computational analyses confirming stable target engagement and anti-proliferative effects in BC models (41, 42).

Moreover, NUDT5 overexpression is implicated in CRC, NSCLC, esophageal squamous cell carcinoma (ESCC), and ccRCC. In CRC, high NUDT5 levels correlate with advanced tumor stages, lymph node metastasis, and reduced post-surgical survival (5). NSCLC and ESCC models demonstrate that NUDT5 silencing suppresses proliferation, migration, and invasion by inactivating MAPK/MEK/ERK pathway, while its upregulation drives metastasis and poor outcomes (18, 20). Contrastingly, low NUDT5 expression predicts unfavorable prognosis in ccRCC, suggesting tissue-specific regulatory roles (43).

In conclusion, NUDT5 is central to cancer cell survival, metabolic adaptation and therapeutic vulnerability. Further validation of its pathway crosstalk and inhibitor efficacy in preclinical models is critical to harness its full potential in precision oncology.

### 2.4 NUDT10 and NUDT11

Canonically, NUDT10 and NUDT11 function as diphosphoinositol polyphosphate phosphohydrolases (DIPP3), enzymes that hydrolyze specialized inositol pyrophosphate signaling molecules. This conserved biochemical role is complemented by a tissue-specific expression pattern established through evolutionary subfunctionalization, with NUDT10 highly expressed in liver, kidney, and testis, and NUDT11 expression largely restricted to the brain (44). Emerging evidence highlights the dual prognostic roles of NUDT10 across cancers. The prognostic significance of NUDT10 exhibits tissue-specific duality. In GC, despite overall downregulation in tumors, higher NUDT10 levels within the tumor cohort are an independent predictor of poor survival (45). Conversely, in prostate cancer, lower NUDT10 expression correlates with adverse outcomes (46). TCGA-based Cox analyses reveal NUDT10 upregulation in ccRCC (47), and it serves as an independent prognostic marker in HCC (48). Both NUDT10 and NUDT11, M7G-associated genes, exhibit tumor-specific regulatory patterns: NUDT11 links to cellular phenotypes in prostate cancer (49), while their combined expression independently stratifies survival risks in bladder cancer (50). These context-dependent roles underscore their potential as tissue-specific biomarkers.

### 2.5 NUDT15 and NUDT16

NUDT15 and NUDT16 critically influence leukemia biology and therapeutic responses. NUDT15 hydrolyzes active 6-thioguanine triphosphate (6-TGTP), modulating thiopurine efficacy and toxicity. NUDT15 variants drive mercaptopurine (MP) intolerance and leukopenia in pediatric acute lymphoblastic leukemia (ALL), mandating genotype-guided dosing to mitigate toxicity (51–53). Pharmacological inhibition (e.g., TH7755, a non-cytotoxic NUDT15 inhibitor) enhances 6-TGTP accumulation, sensitizing malignancies to thiopurines (54, 55). Beyond hematological toxicity, NUDT15 deficiency creates a therapeutic vulnerability in solid tumors with somatic retinoblastoma 1 (RB1) loss, enabling targeted repurposing of thiopurines for precision therapy (56). Clinically, high NUDT15 expression predicts advanced CRC progression and poor post-resection survival (5).

NUDT16 fulfills two key physiological roles in genome maintenance. First, it functions as a (deoxy)inosine diphosphatase that hydrolyzes non-canonical nucleotides such as inosine diphosphate (IDP) and deoxyinosine diphosphate (dIDP), thereby sanitizing the nucleotide pool to prevent their misincorporation (57). Second, it is recruited to DNA double-strand breaks where it removes ADP-ribosylation from 53BP1, a critical post-translational modification that governs 53BP1 protein stability and its proper recruitment to damage sites for repair (58). Beyond these fundamental roles in genomic integrity, NUDT16 also promotes resistance to DNA-damaging therapies by stabilizing HMGA1 via dePARylation, thereby enhancing replication fork stability (59). Its deletion in T-cell acute lymphoblastic leukemia (T-ALL) disrupts RNA degradation, stabilizing oncogenic transcripts to accelerate leukemogenesis (60). While NUDT16 silencing suppresses the proliferation of HeLa cells (57), its overexpression confers radiation resistance, underscoring context-dependent roles in tumorigenesis (61).

These findings establish NUDT15 as both a biomarker for thiopurine precision therapy (62) and a target for adjuvant strategies, whereas NUDT16 vulnerabilities in DNA repair-deficient cancers warrant therapeutic exploitation. Elucidating their crosstalk may refine approaches to circumvent drug resistance in leukemia management.

### 2.6 NUDT20

NUDT20 (Dcp2), a key component of the mRNA decapping complex, plays dual roles depending on the cellular microenvironment: it can either suppress or promote tumor formation. In gliomas, its upregulation enhances proliferation, invasion, and migration while suppressing apoptosis, correlating with poor overall survival and serving as a prognostic biomarker and immunotherapeutic target (63). Conversely, in NSCLC, NUDT20 acts as a tumor suppressor by binding the WAP four-disulfide core domain 21 pseudogene (WFDC21P) to attenuate another carcinogen miR-4293-driven oncogenicity (64). In small cell lung cancer (SCLC), methyltransferase-like 3 (METTL3)-mediated degradation of NUDT20 drives chemoresistance by activating mitophagy (65). The dual role of NUDT20 as both an oncogenic driver and tumor suppressor highlights its cell-type-specific regulatory mechanisms and therapeutic promise.

### 2.7 NUDT21

NUDT21, a central regulator of alternative polyadenylation (APA) (66), exemplifies the profound context-dependency of NUDT, functioning as either an oncogene or a tumor suppressor in a tissue-specific manner. NUDT21 drives tumor progression across several malignancies by regulating distinct sets of target genes. In pancreatic cancer (PC), it promotes proliferation and tumor growth through two identified mechanisms: by binding MZT1 to drive APA-mediated oncogenesis (67), and by enhancing NDUFS2 stability to activate the PI3K/AKT pathway (68, 69). Similarly, NUDT21 upregulation in head and neck squamous cell carcinoma (HNSCC) sustains proliferation by interrupting G2/M cell cycle arrest, while its knockdown induces apoptosis (70). In GC, NUDT21 upregulates SGPP2 to enhance proliferation, migration, and invasion (71). In ovarian cancer, it is recruited by IGF2BP3 to mediate 3’UTR shortening of SPTBN1, promoting the production of an oncogenic short isoform (72). Its oncogenic role extends to B-cell acute lymphoblastic leukemia (B-ALL), where it regulates circBARD1 to activate pro-leukemogenic p38 signaling (73) and to prostate cancer, where its hypomethylation promotes enzalutamide resistance by suppressing docosahexaenoic acid (DHA) biosynthesis (74).

Conversely, NUDT21 acts as a tumor suppressor in a distinct set of cancers, including SCLC, where its depletion accelerates proliferation and inhibits apoptosis (75). In HCC, it suppresses tumorigenesis through dual, complementary mechanisms: it inhibits the expression of oncogenes PSMB2 and CXXC5 via APA (76), and its knockdown impairs circular RNA (circRNA) biogenesis, thereby liberating oncogenic miRNAs (77, 78). In BC, NUDT21 suppresses proliferation, invasion, and EMT via the NUDT21/CPSF6 signaling pathway (79). A similar tumor-suppressive function is observed in kidney renal clear cell carcinoma (KIRC), where its downregulation elevates the oncogene MORC2 (80), and in cervical cancer (CC) and bladder cancers, where NUDT21 loss activates oncogenic Wnt/β-catenin and NF-κB signaling pathways (81, 82). The role of NUDT21 in glioblastoma presents a complex picture, with reported functions as both an oncogene (83) and a tumor suppressor (84). This apparent contradiction likely stems from tumor sub-type heterogeneity and underscores the critical need for context-specific investigation.

In summary, NUDT21 is a pleiotropic regulator whose impact on the cancer phenotype is determined by the tissue-specific transcriptional landscape. Its consistent association with patient prognosis across diverse cancers underscores its clinical relevance as both a robust prognostic biomarker and a promising, albeit complex, therapeutic target.

### 2.8 NUDT22

NUDT22, an emerging NUDT subfamily member, demonstrates unique structural architecture and catalytic specificity toward UDP-glucose and UDP-galactose, driving enhanced glycolysis in malignancies (85). Its overexpression in tumors correlates with aggressive proliferation and unfavorable clinical outcomes. Mechanistically, p53 directly activates NUDT22 transcription by binding its promoter, sustaining elevated expression to circumvent pyrimidine biosynthesis deficits in cancer cells (86). This regulatory axis underscores NUDT22’s pivotal role in metabolic reprogramming and tumor survival, indicating its potential as a therapeutic target in oncological interventions.

### 2.9 Others

Emerging evidence highlights the prognostic and functional diversity of NUDT family members across cancers. In ccRCC, NUDT3, NUDT6, NUDT9P1, NUDT12, NUDT17, and NUDT19 correlate with patient survival, where elevated NUDT17 predicts improved OS (43). In LUAD, NUDT3 is significantly upregulated, correlating with advanced tumor stage and mutational burden, positioning it as a key promoter of disease progression (87). Paradoxically, while NUDT4 is downregulated in ccRCC (43), its upregulation enhances selumetinib efficacy in this malignancy (47). Conversely, NUDT4 exhibits oncogenic properties in LUAD by driving proliferation (88). Although NUDT4 functions as an oncogene in PC due to its elevated expression, circCGNL1 counterintuitively interacts with cytoplasmic NUDT4 to trigger tumor-suppressive apoptosis via the NUDT4/histone deacetylase 4/RUNX family transcription factor 2/guanidinoacetate N-methyltransferase pathway (89). In CRC, NUDT13 acts as a key tumor suppressor by binding to PKM1 and inhibiting its PARP1-mediated ADP-ribosylation, thereby stabilizing PKM1 and promoting a tumor-suppressive OXPHOS phenotype (90). NUDT18 overexpression in CRC clinically correlates with advanced American Joint Committee on Cancer (AJCC) stages and higher T/N classifications, while functionally promoting tumor proliferation. This finding mechanistically validated by suppressed cell growth upon NUDT18 knockdown (5). Notably, NUDT18 exhibits a contrasting tumor-suppressive role in endometrial cancer (EC), where its expression inversely correlates with tumor stage and serves as a favorable prognostic marker (91). NUDT7 downregulation in CRC elevates palmitic acid levels, which activates the Wnt/β-catenin pathway to drive tumorigenesis. Restoring NUDT7 expression suppresses CRC proliferation *in vitro* and *in vivo*, supporting its tumor-suppressive role (92). Beyond somatic expression, germline genetics implicate NUDT17 polymorphisms in BC susceptibility, where specific protective variants significantly reduce disease risk (93). Hypoxia-induced HIF1αstabilization in CC involves NUDT9/NUDT12 knockdown (94), whereas NUDT14 overexpression accelerates rectal carcinogenesis (95). These context-dependent roles, which span metabolic regulation, epigenetic modulation, and hypoxic adaptation, position specific NUDT members as dual-function biomarkers or therapeutic targets.

## 3 Beyond the NUDT family: related hydrolytic enzymes in cancer (e.g., DCTPP1)

While this review focuses on the NUDT family, other structurally distinct but functionally analogous “housekeeping” enzymes are also critical in cancer and represent promising therapeutic targets. A prime example is deoxycytidine triphosphate pyrophosphatase 1 (DCTPP1), a key enzyme responsible for hydrolyzing non-canonical dCTP to maintain nucleotide pool homeostasis and genome stability. Emerging evidence consolidates its considerable role in tumor progression, chemotherapy resistance, and poor prognosis across various cancers, positioning it as a promising nucleotide metabolism-related target for therapy (96). This therapeutic potential is being actively explored. For instance, in CRC, DCTPP1 is overexpressed and associated with poor survival. Crucially, a recently identified natural small-molecule inhibitor was shown to bind directly to DCTPP1, inhibit its enzymatic activity, disrupt metabolic reprogramming, and exert potent anti-tumor effects both *in vitro* and *in vivo*, thereby validating DCTPP1 as a druggable target (97). The parallel biology between DCTPP1 and NUDT enzymes (e.g., NUDT1) underscores a broader therapeutic paradigm: targeting nucleotide pool sanitization pathways represents a generalized vulnerability in cancers with high replication stress. Exploring the crosstalk between these enzyme families could inform effective combination therapies aimed at preventing resistance.

## 4 Future prospects

**Figure 3:**
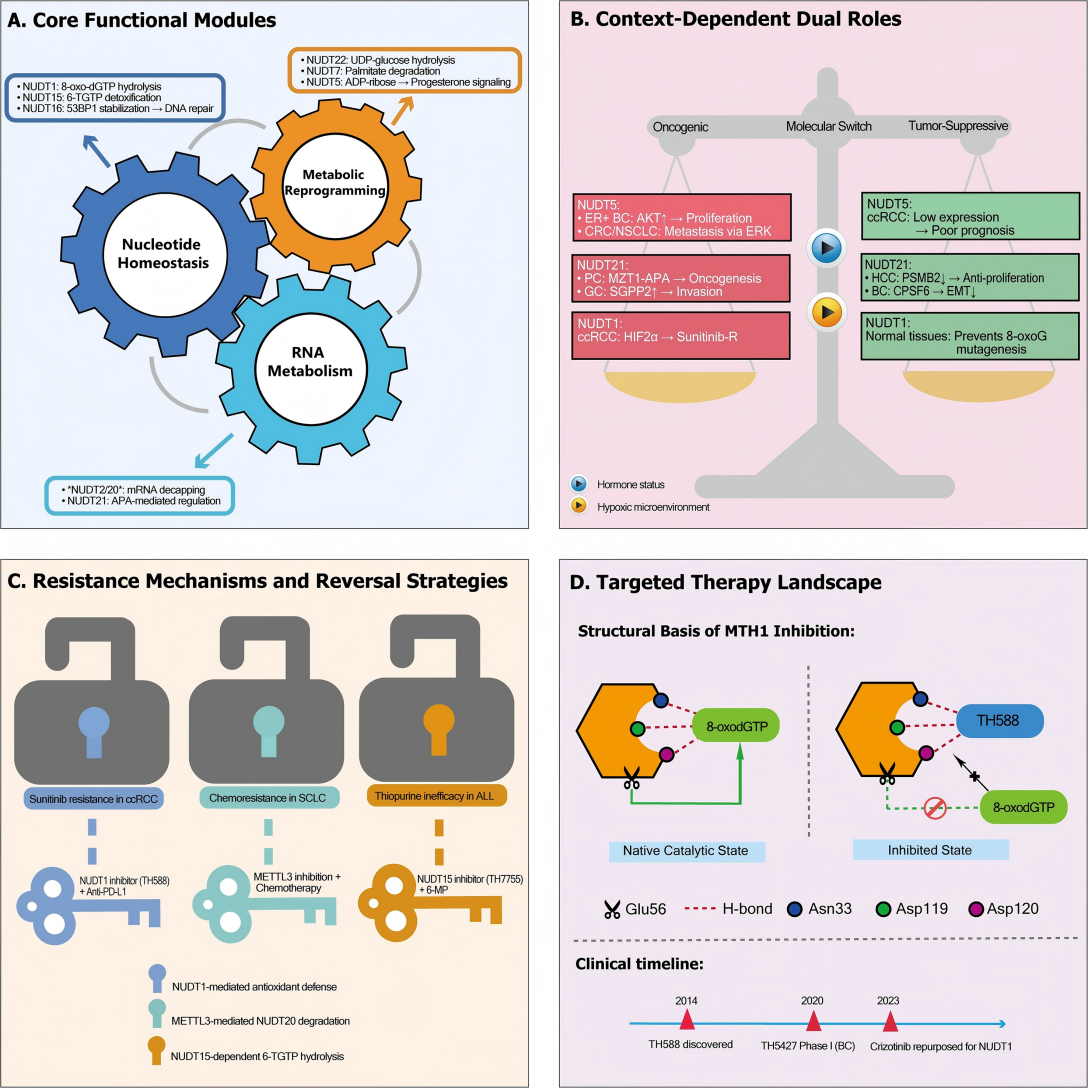
Integrated Networks and Therapeutic Landscape of NUDT in Cancer. **(A)** Core functional modules. NUDT hydrolases drive oncogenesis through three interconnected modules: Nucleotide Homeostasis (e.g., NUDT1 hydrolyzes 8-oxo-dGTP; NUDT15 detoxifies 6-TGTP; NUDT16 stabilizes 53BP1 for DNA repair), RNA Metabolism (e.g., NUDT2/20 mediate mRNA decapping; NUDT21 regulates APA to control gene expression), and Metabolic Reprogramming (e.g., NUDT22 hydrolyzes UDP-glucose; NUDT7 degrades palmitate to suppress Wnt/β-catenin; NUDT5 hydrolyzes ADP-ribose, impacting progesterone signaling). **(B)** Context-dependent dual roles. Selected NUDTs exhibit tissue-specific oncogenic (left, red) or tumor-suppressive (right, green) functions, influenced by molecular switches such as hormone status and hypoxic microenvironment. Examples include the oncogenic role of NUDT5 in ER+ breast cancer (via AKT) and CRC/NSCLC (via ERK), versus its tumor-suppressive association in ccRCC; and the opposing roles of NUDT21 in pancreatic cancer (pro-oncogenic) versus hepatocellular carcinoma (anti-proliferative). **(C)** Resistance mechanisms and reversal strategies. Three paradigms of NUDT-mediated therapy resistance and their targeted countermeasures: NUDT1 confers sunitinib resistance in ccRCC via antioxidant defense, reversed by combining TH588 with anti-PD-L1 antibody; METTL3-mediated degradation of NUDT20 drives chemoresistance in SCLC, reversed by METTL3 inhibition; NUDT15 hydrolyzes 6-TGTP leading to thiopurine inefficacy in ALL, counteracted by the inhibitor TH7755. **(D)** Targeted therapy landscape. Molecular basis of NUDT1 inhibition. (Left panel) Active State with Bound Substrate: The native catalytic conformation of MTH1 with its natural substrate, 8-oxodGTP, bound in the active site. The substrate is positioned for hydrolysis by the catalytic residue Glu56. (Right panel) Competitive Inhibition by TH588: The inhibitor TH588 occupies the substrate-binding site, forming key hydrogen bonds with Asn33, Asp119, and Asp120. This occupancy spatially blocks access to the catalytic residue Glu56, rendering it functionally inaccessible and preventing substrate turnover. (Bottom) Clinical development timeline shows key milestones from TH588 discovery (2014) to crizotinib repurposing (2023).

The multifaceted roles of NUDT in cancer, synthesized in **Figure 3**, argue for a paradigm shift: from viewing them as isolated metabolic enzymes to targeting them as components of an adaptive oncogenic network. This network, composed of three cores, interacting modules, which include nucleotide homeostasis, RNA metabolism, and metabolic reprogramming (**Figure 3A**), enables tumors to maintain genomic integrity, dynamically regulate oncogenic gene expression, and fuel rapid proliferation. The therapeutic challenge and opportunity lie in the network’s context-dependent duality (**Figure 3B**), where the same NUDT member can be a driver in one tissue (e.g., NUDT5 in BC) and a suppressor in another (e.g., NUDT5 in ccRCC). This duality is dictated by molecular switches like specific hormone receptor status or hypoxia, mandating that therapeutic strategies be informed by precise biomarkers to exploit cancer-specific vulnerabilities while sparing normal functions.

A critical barrier to effective therapy is the intrinsic or acquired resistance mediated by this network. **Figure 3C** models this as tractable biological problems: NUDT1 antioxidant activity conferring resistance to targeted agents like sunitinib, RNA metabolism rewiring (via METTL3-NUDT20 axis) leading to chemoresistance, and NUDT15-mediated inactivation of thiopurines. The “lock-and-key” illustration emphasizes that rational combination therapies, such as pairing NUDT inhibition with immunotherapy, chemotherapy, or complementary targeted agents, can overcome these resistance mechanisms.

The translation of these insights into clinical practice is guided by an evolving pharmacological landscape, as summarized in **Figure 3D**. Structural elucidation of the NUDT1-inhibitor complex reveals that compounds like TH588 achieve potency by binding directly within the nucleotide pocket, forming key hydrogen bonds with Asn33, Asp119, and Asp120, thereby blocking substrate access and inhibiting the essential residue Glu56. These structural insights inform rational drug design. Crucially, the clinical translation of NUDT-targeting strategies must account for a wide therapeutic index. This is exemplified by two contrasting scenarios: patients carrying NUDT15 variants represent a high-risk population for thiopurine toxicity, necessitating genotype-guided dose reduction, whereas tumors with high NUDT22 expression present a high-opportunity context for targeting glycolytic dependencies. The integrated timeline encapsulates this paradigm shift, chronicling the key milestones from the initial discovery of NUDT1 inhibitors to the strategic repurposing of existing drugs like crizotinib. This trajectory underscores the field’s evolution from target identification towards sophisticated translational and combinatorial strategies.

To navigate this complex landscape and realize the clinical potential of targeting NUDT, future efforts must converge on several fronts:

1. Precision Inhibitor Development: Leveraging structural insights (as in **Figure 3D**) to design next-generation, isoform- or context-selective inhibitors, and exploring modalities like proteolysis-targeting chimeras (PROTACs) for targeted degradation.
2. Combinatorial Strategy Mapping: Using functional genomics screens to systematically identify synthetic lethal interactions and optimal drug combinations that prevent resistance, moving beyond empirical pairing.
3. Advanced Biomarker Integration: Validating and deploying biomarkers, such as genetic variants (e.g., NUDT15), expression signatures (e.g., NUDT22-high), and microenvironmental features (e.g., hypoxia), to guide patient selection in clinical trials.
4. Technology-Enabled Discovery: Applying spatial transcriptomics to understand NUDT roles in tumor microenvironment niches, and utilizing AI for the discovery of allosteric inhibitors or novel chemical matter.
5. Expanding the Therapeutic Horizon: Investigating the immunomodulatory potential of RNA metabolism regulators (e.g., NUDT20) and exploring the broader family of nucleotide-sanitizing enzymes (e.g., DCTPP1) for complementary vulnerabilities.

By adopting this integrated framework, which connects molecular mechanism, contextual function, resistance biology, and pharmacological precision, the targeting of NUDT can mature from a promising concept into a cornerstone of personalized cancer therapy.

## 5 Conclusion

In conclusion, the NUDT family drives oncogenesis not as isolated actors, but through an integrated, context-dependent network that interconnects nucleotide homeostasis, RNA metabolism, and metabolic reprogramming. This review reframes our understanding of these enzymes from mere metabolic ‘housekeepers’ to dynamic, environment-sensitive regulators of cancer phenotype. It is precisely this functional plasticity that presents both the greatest challenge and the most promising opportunity for therapeutic intervention. Future efforts must therefore move beyond isoform-specific inhibition towards spatiotemporally resolved targeting, exploiting cancer-specific contextual vulnerabilities while preserving their physiological roles. This paradigm shift will be crucial for translating the complex biology of NUDTs into effective, personalized oncology strategies.
